# Progress of non-small-cell lung cancer with *ROS1* rearrangement

**DOI:** 10.3389/fmolb.2023.1238093

**Published:** 2023-12-22

**Authors:** Xin Yang, Zhe Tang, Jing Li, Jizong Jiang, Yue Liu

**Affiliations:** ^1^ Department of Oncology, Tongji Hospital, Tongji Medical College, Huazhong University of Science and Technology, Wuhan, Hubei, China; ^2^ Department of Thoracic Surgery, Tongji Hospital, Tongji Medical College, Huazhong University of Science and Technology, Wuhan, Hubei, China; ^3^ Department of Thyroid and Breast Surgery, Tongji Hospital, Tongji Medical College, Huazhong University of Science and Technology, Wuhan, China

**Keywords:** *ROS1* rearrangement, fusion gene, tyrosine kinase inhibitor, drug resistance, non-small-cell lung cancer

## Abstract

*ROS1* rearrangement is found in 0.9%–2.6% of people with non-small-cell lung cancers (NSCLCs). Tyrosine kinase inhibitors (TKIs) target *ROS1* and can block tumor growth and provide clinical benefits to patients. This review summarizes the current knowledge on *ROS1* rearrangements in NSCLCs, including the mechanisms of *ROS1* oncogenicity, epidemiology of *ROS1*-positive tumors, methods for detecting rearrangements, molecular characteristics, therapeutic agents, and mechanisms of drug resistance.

## 1 Introduction

Lung cancer remains the most fatal malignant tumor, with approximately 85% of cases being non-small-cell lung cancer (NSCLC). Of those with NSCLC, about 25% carry positive-driven gene changes that can benefit from the corresponding molecular-targeted therapy (Siegel et al., 2022). Compared with epidermal growth factor receptor (*EGFR*) mutations and anaplastic lymphoma kinase (*ALK*) rearrangements, the genetic proto-oncogene tyrosine-protein kinase-1 (*ROS1*) is less prevalent in NSCLC, accounting for approximately 0.9%–2.6% of cases (Bergethon et al., 2012; Cai et al., 2013). The results of prospective Phase I/II clinical trials have confirmed the effectiveness of crizotinib in *ROS1*-positive NSCLC (Shaw et al., 2019a), and in recent years, several targeted drugs, including entrectinib, ceritinib, and lorlatinib, have also shown excellent antitumor activity (Shaw et al., 2017; Peters et al., 2020; Dziadziuszko et al., 2021). This article provides an overview of the progress regarding research on NSCLC with the *ROS1* rearrangement.

## 2 *ROS1* gene

The *ROS1* gene was discovered in the 1980s in the products of bird myeloma virus RNA UR2 (Balduzzi et al., 1981). The human *ROS1* gene is located on chromosome 6q21 (Nagarajan et al., 1986), which belongs to the family insulin receptor genes of receptor tyrosine kinases (RTKs), which encode intermembrane proteins consisting of 2,347 amino acids, including hydrophobic extracellular domains, a transmembrane region, and intracellular parts of the tyrosine kinase domain (Roskoski, 2017). Rikova et al. first reported the role of the *ROS1* oncogene in NSCLC in 2007 and identified two new protein fusion transcription factors, *SLC34A2* and *CD74* (Rikova et al., 2007). With the continuous improvement in modern sequencing technology, an increasing number of fusion species have been discovered, and their role as carcinogenic genes in multiple cancers has been gradually confirmed (Dagogo-Jack et al., 2019; Zhang et al., 2019).

**FIGURE 1:**
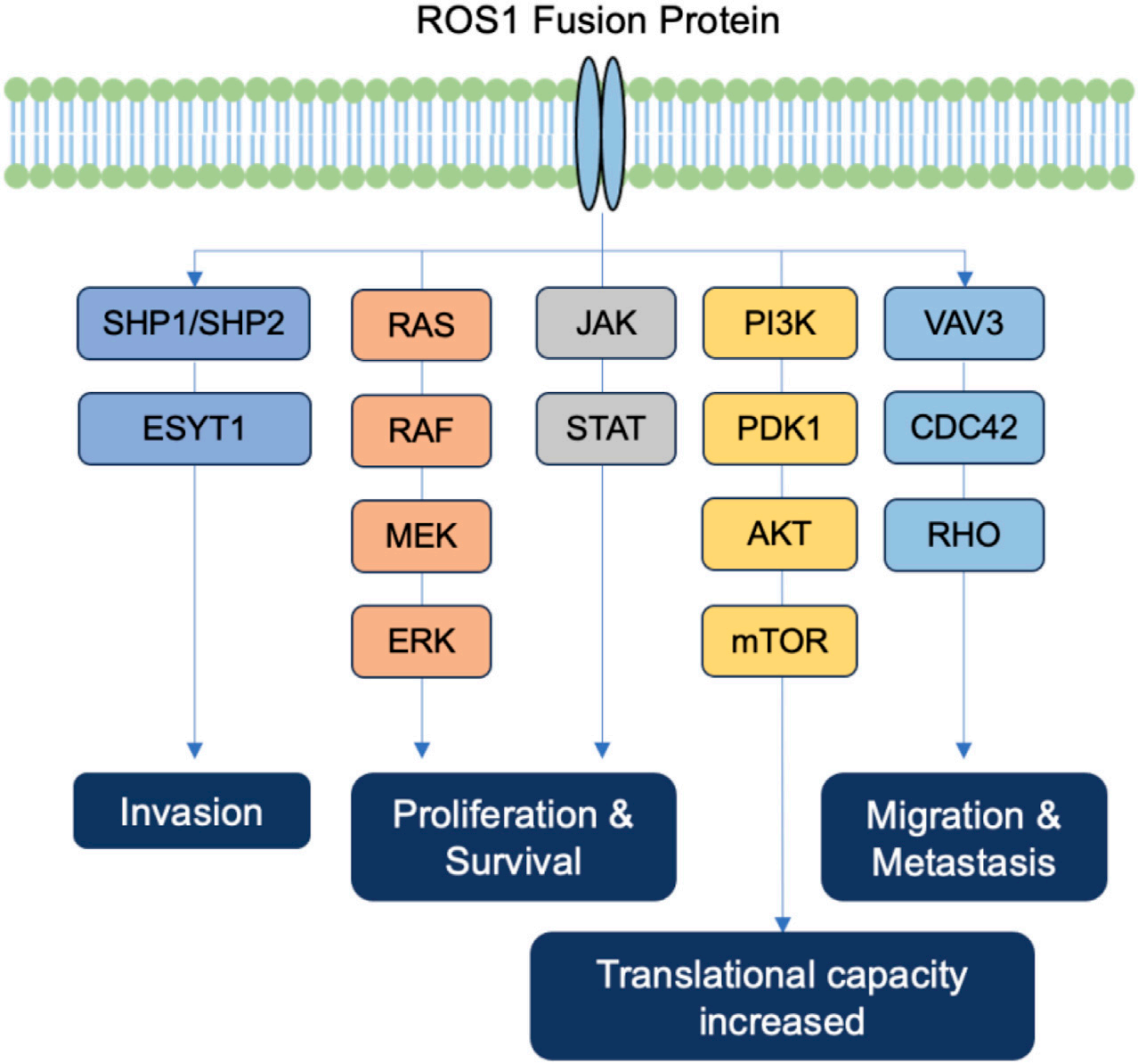
The ROS1 signaling pathway.

*ROS1* plays an important role in activating multiple signaling pathways, including those involved in cell differentiation, proliferation, growth, and survival (Figure 1). *ROS1* rearrangement causes disorders in enzyme-active proteins and the abnormal activation of the associated signaling pathways by forming phosphate thyroxine recruitment spots at the end of the ROS, including tyrosine phosphatase tumor suppressor SHP1/SHP2, pro-mitotic protein extracellular-signal-regulated kinase (ERK)-1/2, insulin-receptor substrate (IRS)-1, phosphatidylinositol 3-kinase (PI3K), protein kinase B (AKT), mitogen-activated protein kinases (MAPKs), signal transducer and activator of transcription (STAT)-3, and the VAV3-related signaling pathway (Huang et al., 2020).

## 3 Epidemiological and clinical features

Among the people with NSCLC in China, approximately 2.59% carry the *ROS1* fusion gene, and approximately 17,000 new cases of *ROS1*-positive NSCLC are estimated to occur annually in China. *ROS1* rearrangements are more common in young, female, and nonsmoking patients (Fu et al., 2015; Zhu et al., 2015). The main pathological types are adhesive, vesicular, or solid glandular cancers; a few are squamous-cell, multicellular, or large-cell cancer (Park et al., 2019), more than 90% of which express thyroid transcription factor-1 (TTF-1), mostly diagnosed as phase III–IV, high incidence of brain transfusions (Drilon et al., 2021). Compared to other types of NSCLC, ROS1-positive NSCLC has a significantly increased risk of developing thromboembolic diseases (Shah et al., 2021; Woodford et al., 2021; Zhu et al., 2021), but the underlying mechanism remains unclear.

## 4 Molecular characteristics

### 4.1 Fusion partners

The most common fusion partners of the *ROS1* gene include *CD74* (38%–54%), *EZR* (13%–24%), *SDC4* (9%–13%), and *SLC34A2* (5%–10%) (Cui et al., 2020; Huang et al., 2021a). With the continuous improvement in DNA- and RNA-sequencing technology, new fusion partners have been discovered, such as *CCCKC6, TFG, SLMAP, MYO5C, FIG, LIMA1, CLTC, GOPC, ZZCCHC8, CEP72, MLL3, KDELR2, LRIG3, MSN, MPRIP, WNK1, SLC6A17, TMEM106B, FAM135B, TPM3,* and *TDP52L1* (Li et al., 2018). People with NSCLC with the *CD74–ROS1* rearrangement have longer progression-free survival (PFS) and overall survival (OS) than those with other types of rearrangements (Li et al., 2018), but no similar conclusions have been found in other studies (Cui et al., 2020). Currently, the relationship between the fusion partners and prognosis remains unclear.

### 4.2 Co-occurring genetic mutations

Approximately 36% of people with NSCLC with *ROS1* rearrangements have co-occurring genetic mutations. Those with co-mutation have a worse prognosis than non-co-mutant patients (PFS 8.5 months versus 15.5 months, *p* = 0.0213) (Zeng et al., 2018). *ROS1* rearrangement typically does not simultaneously occur with mutations in other genes (such as *EGFR, ALK*, and *KRAS*). However, in recent years, occasional cases of *ROS1* rearrangements associated with mutations in other driving genes have been reported (Zhu et al., 2016; Uguen et al., 2017). Lambros et al. reported 15 cases of NSCLC with an *ROS1* mutation in conjunction with an *EGFR* mutation, including 9 cases of 19 deletion, 5 cases of L858R mutation, and 1 case of 20 insertion. *ROS1* rearrangement may be one of the mechanisms of resistance to EGFR inhibitors, and the combination of EGFR inhibitors with crizotinib may be an effective treatment (Lambros et al., 2018). Crizotinib, as a tyrosine kinase inhibitor, binds to the ATP-binding site of the *ROS1* kinase domain, inhibiting its enzymatic activity. By blocking *ROS1* signaling, crizotinib disrupts the intracellular pathways that drive cancer cell proliferation and survival.

Co-mutations in both ROS1 and ALK are rare but occasionally reported. Both mutations were found to be sensitive to crizotinib, which may be a more suitable treatment option. Uguen et al. and Song et al. respectively reported a lung adenocarcinoma patient with concurrent ALK/ROS1 rearrangements confirmed by fluorescence *in situ* hybridization (FISH) analyses, and both patients showed response to crizotinib (Song et al., 2017; Uguen et al., 2017).

*ROS1* and *KRAS* co-mutations are rare and can be primary or successive. In a study involving six patients with the *KRAS–ROS1* comutation, only one patient benefitted from crizotinib treatment, while KRASG13D- or G12V-mutation carriers showed no response (Lin et al., 2017).

*ROS1–MET* comutations are extremely rare. Tang et al. reported one *ROS-1–MET* co-mutation in a series of 15 patients, however, no treatment information was provided (Tang et al., 2018). Rihawi et al. noted one case of NSCLC with a *ROS1–MET* co-mutation, and MET inhibitor capmatinib treatment failed, followed by crizotinib treatment, and a PFS of up to 11 months (Rihawi et al., 2018). Zeng et al. reported a case of NSCLC with *ROS1* rearrangement and MET expansion, with disease progression occurring 1.5 months after crizotinib treatment (Zeng et al., 2018). Therefore, the combined use of highly selective MET inhibitors based on crizotinib treatment is necessary for patients with this type of comutation. Therefore, further clinical research and practice are required.

Comutations in *ROS1* and *BRAF* have been reported, but no reports on combined treatment with an *ROS1* inhibitor with *BRAF* inhibitors have been published (Wiesweg et al., 2017). Furthermore, it was reported that comutations with *TP53* may be associated with a shorter survival time (Lindeman et al., 2018) (Table 1).

**TABLE 1:** Summary of co-occurring genetic mutation of ROS1.

Co-occurring genetic mutation of ROS1	Effect
*ROS1-EGFR*	ROS1 rearrangement may be one of the mechanisms of resistance to EGFR inhibitors; Combination of EGFR inhibitors with crizotinib may be effective
*ROS1-ALK*	Both mutations are sensitive to crizotinib, suggesting crizotinib as a potential treatment
*ROS1-KRAS*	Limited response to crizotinib; Variable treatment outcomes
*ROS1-MET*	Variable treatment outcomes; Combined use of highly selective MET inhibitors with crizotinib is necessary
*ROS1-BRAF*	Limited information on treatment outcomes; No published reports on combined treatment with ROS1 and BRAF inhibitors
*ROS1-TP53*	Associated with a shorter survival time

## 5 Methods of *ROS1* detection

The detection of *ROS1* is currently recommended in all patients with non-squamous-cell cancer to determine the indication for the selection of the corresponding targeted drug.

Fluorescence *in situ* hybridization (FISH) is the gold standard for diagnosing *ROS1* rearrangements. FISH uses a two-probe (3′ and 5′) separation design and can detect ≥50 tumor cells; the result is considered positive when more than 15% of the cells show 3′- and 5′-probe separation or separate 3′ signals. The disadvantages of FISH include its high cost, technical difficulty, and time consumption. At present, probes that can detect both *ROS1* and *ALK* rearrangements have been put into clinical use as they have less strict requirements for tumor samples (Ginestet et al., 2018; Zito Marino et al., 2020).

The immunohistochemistry (IHC) technique is commonly used to screen for *ROS1* arrangements owing to its convenience, low cost, and ease of operation; IHC has a sensitivity of approximately 90%–100% and a specificity of approximately 70%–90%. False-positive outcomes may occur in one-third of patients, especially in those with adhesive or adenomatous EGFR-mutant glandular cancer (Wang et al., 2020; Makarem et al., 2021). Therefore, positive or suspicious IHC results require further confirmation by FISH, reverse -transcription quantitative polymerase chain reaction (RT-qPCR), or next-generation sequencing (NGS) (Hofman et al., 2019; Cheung et al., 2021; Fielder et al., 2022).

Two situations should be noted: 1) IHC positivity and FISH negativity, indicating the presence of another carcinogen-driven mutation not included in FISH detection and requiring further confirmation by RT-qPCR or NGS testing (Kim et al., 2021); 2) FISH may result in false-negative results for some fusion partners, mainly *GOPC–ROS1* or *EZR–ROS1*. In the latter, the 5′-*ROS1* gene is usually lacking, and the corresponding FISH detection uses a separate 3′ probe (Capizzi et al., 2019). In a recent study, the authors detected a rare case of NSCLC with *ROS1* fusion (*SQSTM1*), *ROS1* mutation, and *ROS1* expansion with positive IHC expression using NGS technology (Huang et al., 2021b). Therefore, if the results remain uncertain after both IHC and FISH testing, NGS should be performed to confirm the presence of unusual fusion genotypes.

With RT-qPCR, the unique primers detecting *ROS1* rearrangements are used; the method has a sensitivity of up to 100% and a specificity of 85.1%. The disadvantage of the method is its technical difficulty, requiring several steps, including RNA extraction, complementary DNA synthesis, quantitative PCR, and data analysis, which are currently more commonly applied in laboratories: few clinical applications are lacking (Shan et al., 2015).

NGS can be used to theoretically identify all fusion partners, including new variation types and other carcinogenic genetic variations. The requirements for the samples are no strict, and tumor tissues or blood plasma can be used as test samples. Recent advances in NGS technology for detecting ROS1 rearrangements involve both DNA and RNA approaches. DNA-based methods, such as targeted sequencing, whole exome sequencing (WES), and whole genome sequencing (WGS), target specific genomic regions or the entire genome to identify structural alterations. RNA-based techniques, including targeted RNA sequencing and RNA-Seq, directly detect fusion transcripts, providing valuable information on gene rearrangements. Hybrid approaches integrating DNA and RNA analyses enhance sensitivity and specificity. Improved bioinformatics tools and the use of single-molecule sequencing technologies contribute to increased accuracy, while emerging liquid biopsy methods offer less invasive options. Combining these approaches allows for a comprehensive and precise assessment of ROS1 rearrangements in lung cancer genomes (Clave et al., 2019). NGS was used to detect many novel uncommon ROS1 fusions, most of which were reported to be sensitive to matched targeted therapy, similar to the canonical fusions. The clinical significance of some genomic breakpoints remained unclear and could be explored further via NGS technology (Li et al., 2022). The disadvantages are that the cost is higher and the results cannot be quickly obtained (Mosele et al., 2020).

## 6 Treatment of NSCLC with *ROS1* rearrangement

The U.S. Food and Drug Administration (FDA) has approved crizotinib and entrectinib as first-line treatments for patients with unresectable NSCLC with *ROS1* rearrangements. Other tyrosine kinase inhibitors (TKIs), including ceritinib and lorlatinib, also exhibit excellent antitumor activity.

### 6.1 Crizotinib

Crizotinib is a small molecule inhibitor with a complex molecular structure. It contains various functional groups, including pyrazole, pyridine, and piperidine rings. The three-dimensional structure allows it to bind to the ATP-binding site of the ROS1 kinase domain, inhibiting its activity. The Phase I PROFILE 1001 study included 50 people with *ROS1*-positive advanced NSCLC receiving crizotinib (250 mg twice daily). The objective remission rate (ORR) was 72%; disease control rate (DCR) was 90%; median duration of response (DOR) was 24.7 months; and median PFS and OS were 19.3 months and 51.4 months, respectively. The most common adverse events included visual impairment (82%), diarrhea (44%), nausea (40%), edema (40%), constipation (34%), vomiting (34%), elevated transaminase (22%), fatigue (20%), and taste disturbance (18%). Most adverse reactions were grade 1 to 2^4^.

Compared with the PROFILE 1001 study, in the AcSé Phase I/II study based on 37 patients, the effectiveness of crizotinib was relatively poor, with an ORR of 47.2%, and median PFS and OS of 5.5 and 17.2 months, respectively. The researchers presumed that more patients with a higher performance status (PS) score of two points (25% vs. 2%) were grouped in the AcSé study (Wu et al., 2018). The results of the EUCROSS and METROS studies from Europe were similar to those of the PROFILE 1001 study, with ORRs of 70% and 65%, and median PFS values of 20 and 22.8 months, respectively (Michels et al., 2019).

Although crizotinib showed excellent anti-tumor activity in the treatment of *ROS1*-positive NSCLC, its blood–brain barrier penetration rate was low, and brain metastases (47%) became the main area of disease progression. In addition, approximately 36% those with *ROS1*-positive NSCLCs also experienced brain metastases at the baseline level. Therefore, ROS1–TKIs that can better cross the blood–brain barrier should be developed in the future.

### 6.2 Entrectinib

Entrectinib is a multitarget inhibitor of ROS1, ALK, and pan-tropomyosin receptor kinase (TRK). Its molecular structure consists of various cyclic and aromatic structures, including a tetrahydropyrrolopyrazine ring. Entrectinib is designed to penetrate the blood-brain barrier, making it effective against central nervous system metastases. In *in vitro* experiments, its anti-ROS1 activity was 40 times stronger than that of crizotinib (Rolfo et al., 2015). The results of two Phase I/II studies showed antitumor activity and good tolerance to entrectinib (Drilon et al., 2017). The most common side effects included discomfort in taste (41.4%), fatigue (27.9%), dizziness (25.4%), constipation (23.7%), diarrhea (22.8%), nausea (20.8%), and weight gain (19.4%). The results of the STARTRK-2 study confirmed the efficacy of entrectinib in *ROS1*-positive NSCLC involving a total of 161 patients who had not previously received anti-*ROS1* treatment. Of these, 34.8% people also experienced baseline brain metastases. The ORR was 67.1%, PFS median was 15.7 months, and 1-year OS rate was 81%. For 24 patients at the baseline with measurable brain metastases, the intracerebral ORR was 79.2%, and intracerebral PFS was 12 months (Drilon et al., 2020).

### 6.3 Lorlatinib

Lorlatinib is a third-generation *ROS1* inhibitor designed to overcome resistance mutations that may develop during treatment with earlier-generation inhibitors. It has a more intricate structure compared to crizotinib, with multiple fused rings and functional groups, increasing the permeability of the blood–brain barrier by reducing P-glucose-1-mediated exudation. Lorlatinib exhibits activity against *ROS1* as well as *ALK*. In a Phase I/II study, 61 people with *ROS1*-positive NSCLC were included, including 21 patients treated with primary TKIs and 40 patients previously treated with crizotinib. The ORR of the primary TKI patients was 62%, their median PFS was 21 months, their intracerebral ORR was 64%, and intracerebral PFS was not achieved. The ORR, median PFS, and intracerebral ORR of the patients treated with crizotinib were 35%, 8.5 months, and 50%, respectively. The most common adverse events included hypocholesterolemia (65%), hypoglycemia (42%), peripheral edema (39%), surrounding neuropathy (35%), cognitive changes (26%), weight gain (16%), and mood disorders (16%). The incidences of grade 3 and 4 adverse reactions were 43% and 6%, respectively (Shaw et al., 2019b). Monitoring the plasma concentration of lorlatinib may help control adverse events without altering the effectiveness of this antitumor therapy (Chen et al., 2021). When resistance mutations, such as *ROS1* ^*K1991E*^ or *ROS1* ^*S1986F*^, appear after treatment with crizotinib, lorlatinib may have a stronger effect; however, it has a minor effect on the resistance mutation type *ROS1* ^*G2032R*^.

### 6.4 Ceritinib

Ceritinib is an ALK inhibitor that exhibits antitumor activity and intracerebral effects in those with ALK-positive NSCLC (Shaw et al., 2017). The molecular structure of ceritinib includes various aromatic rings and functional groups. *In vitro* experiments showed that ceritinib has potential anti-*ROS1* rearrangement activity. In a Phase II study involving 32 patients with *ROS1*-positive NSCLC, the ORR for ceritinib treatment was 62%, and the median PFS was 9.3 months. In the subgroup (*n* = 30) that was not treated with crizotinib, the median PFS was 19.3 months, and its effectiveness was comparable to that of other TKIs. The ORR in the brain metastasis subgroup (*n* = 8) was 63%. Its tolerance was similar to that of other TKIs, with an incidence of 37% of adverse events of grade 3 or above (Drilon et al., 2016).

### 6.5 Cabozantinib

Cabozantinib is a small-molecule TKI and consists of a pyridine ring with a fluorine atom and a 3-(morpholin-4-yl) propoxy group attached to it, as well as a 3-aminopyridine-2-carboxamide group and a 4-(6-(propan-2-yl) pyridin-3-yl) benzoic acid group (Maroto et al., 2022). It targets ROS1, MET, VEGFR-2, RET, and AXL and has a strong ability to penetrate the blood–brain barrier. The results of preclinical studies and case reports have indicated that cabozantinib is effective for treating patients with resistance to other TKIs (crizotinib, entrectinib, and ceritinib) and resistance mutations (such as *D2033N* or *G2032R*) (Sun et al., 2019). Therefore, cabozantinib is clinically used as a treatment option after the development of resistance to other TKIs.

### 6.6 Brigatinib

Brigatinib is a kinase inhibitor with a complex structure. It contains pyrimidine, pyridine, and aniline moieties, among others, arranged in a way that allows it to inhibit the activity of certain tyrosine kinases, it is a multitarget inhibitor of ROS1 and ALK and has antitumor activity against EGFR-mutant NSCLC. Brigatinib exhibits antitumor activity against several drug-resistant *ROS1* mutations. In one study, researchers assessed the efficacy and tolerance of brigatinib in eight patients with *ROS1*-positive NSCLC, one of whom did not receive TKI treatment, and seven of whom developed disease progression after crizotinib treatment (Dudnik et al., 2020). The ORRs for the total population and the crizotinib-treated subgroups were 37% and 29%, respectively. No Grade 3 or 4 adverse events were observed. In one case report, the disease progressed after prior treatment with several ROS1-TKIs, and brigatinib therapy remained effective (Hegde et al., 2019). *In vitro* experiments showed that Bugatti has strong anti-tumor activity against NSCLC carrying the *L2026M* mutation but is ineffective against the *G2032R* mutation (Camidge et al., 2018).

### 6.7 Repotrectinib

Repotrectinib (TPX-0005) is also a multitarget TKI that can target ROS1, TRK, and ALK and effectively cross the blood–brain barrier. It contains various functional groups, including cyclic structures and heteroatoms like nitrogen and oxygen. The specific arrangement of atoms in Repotrectinib allows it to interact with the ATP-binding sites of these kinases, inhibiting their activity and disrupting the signaling pathways that contribute to cancer cell growth. In preclinical studies, repotrectinib showed strong antitumor activity in NSCLC models with *ROS1*-positive brain metastases, prior ROS1-TKI treatment, and *ROS1* ^*G2032R*^ mutations (Yun et al., 2020). A Phase I/II clinical trial (NCT03093116) to evaluate the safety and efficacy of repotrectinib in ROS1-positive NSCLC is currently underway.

### 6.8 Taletrectinib

Taletrectinib (DS-6051B) is an inhibitor targeting *ROS1* and *NTRK* and shows antitumor activity against crizotinib-resistant NSCLC, including in patients carrying the *G2032R* mutation. In *in vitro* experiments, taletrectinib showed strong activity against resistance mutations, such as *G2032R, L1951R, S1986F*, and *L2026M*. Two Phase I clinical trials conducted in the United States and Japan evaluated the effectiveness of taletrectinib in patients with *ROS1*-positive NSCLC. The former included 46 patients with an ORR of 33% in patients with crizotinib resistance; the latter included 15 patients with ORRs of 58.3% and 66.7% in all patients and in patients with no prior crizotinib treatment, respectively (Papadopoulos et al., 2020).

### 6.9 Ensartinib

Ensartinib is an ALK-TKI that demonstrated 10-times higher anti-ALK activity than crizotinib in *in vitro* experiments. A Phase II trial of *ROS1*-positive NSCLC (NCT03608007) showed a certain therapeutic effectiveness, with an ORR of 27%, and intracerebral disease control was achieved in three out of four patients with brain metastases (Ai et al., 2021).

### 6.10 Other treatments

Chemotherapy remains the recommended second-line treatment after the failure of crizotinib treatment. The combination of antivascular therapy with ROS1-TKIs is another potentially effective treatment strategy. In *in vitro* experiments, the combined use of vascular endothelial growth factor (VEGF) blockers and ROS1-TKI increased antitumor activity (Watanabe et al., 2021). In a clinical trial involving 14 patients, those with NSCLC that was *ALK*-positive, *ROS1*-positive, or had *MET* expansion showed an ORR was 58.3% with good tolerance; however, 3 patients discontinued treatment due to hepatotoxicity or hemorrhage (Saito et al., 2019).

The effectiveness of immunotherapy in *ROS1*-positive NSCLC has not yet been fully elucidated. In *in vitro* experiments, ROS1 was found to regulate the expression of programmed death protein ligand-1 (PD-L1) by activating the MEK–ERK and ROS1–SHP2 signaling pathways. Most ROS1-positive tumors do not express PD-L1 and have a low tumor mutation burden (TMB) (Choudhury et al., 2021). The results of small-sample studies have shown that the ORR of people with *ROS1*-positive NSCLC receiving mono-immunotherapy was 13%–17% (Mazieres et al., 2019), and the ORR of immunotherapy combined with chemotherapy was 83% (Guisier et al., 2020). Choudhury et al. (Choudhury et al., 2021) found no noticeable differences in the expression levels of PD-L1 between patients who received effective and ineffective immunotherapies. In patients with TKI resistance, combined immunotherapy has a high clinical application value, but potential toxic reactions to sequential immunotherapy with TKIs must be monitored.

## 7 Resistance mechanisms of ROS1 inhibitors

### 7.1 Resistance mechanisms of crizotinib

#### 7.1.1 Structural domain mutations

ROS1 kinase structural domain mutation is the most common resistance mechanism to crizotinib, accounting for approximately 40%–55% of the total. G2032R is the most common type of mutation, occurring in the solvent area of the ATP binding site, accounting for approximately 33%–41% of cases (Awad et al., 2013). In *in vitro* studies, the *ROS1* ^*G2032R*^ mutation increased the expression of *TWIST1*, promoting epithelial–mesenchymal transition (EMT), cell migration, and resistance to ROS1-TKIs by modifying the combination of location points and spatial blockages (Gou et al., 2018). Currently, repotrectinib, topotrectinib, and cabozantinib have shown improved anti-ROS1^G2032R^ activity (Gou et al., 2018).

Other common mutations include *D2033N* (2.4%–6%), *S1986Y*/F (2.4%–6%), *L2026M* (1%), *L2155S* (1%), *L1951R* (1%), and *S1886* (1%) (Katayama et al., 2015) (Table 2). *ROS1* ^*D2033N*^ induces the modification of ATP-binding pockets, resulting in the weakening of the ability of tumor cells to bind to ROS1-TKIs. In *in vitro* experiments, *ROS1* ^*D2033N*^ led to resistance to crizotinib, entrectinib, and ceritinib, but remained sensitive to lorlatinib, repotrectinib, and cabozantinib. *ROS1* ^*S1986F*^ results in resistance to crizotinib, entrectinib, and ceritinib by changing the position of a ring structure rich in glycine at the end of the C spiral (Facchinetti et al., 2016).

**TABLE 2:** Common mutation types of crizotinib resistance and effective inhibitors.

Type of ROS1 fusion	Mutation site	Mechanisms	Effective TKI
CD74-ROS1	G2032R	Altered spatial structure of ROS1 domain interferes with drug binding and leads to resistance to ROS1 inhibitors	cabozantinib
			repotrectinib
	D2033N	Alteration in the electrostatic force on the outer surface of the ATP-binding site and a rearrangement of ATP-binding site	lorlatinib
			cabozantinib
			repotrectinib
	L2026M	Leucine to methionine substitution	ceritinib
			lorlatinib
			cabozantinib
			repotrectinib
SLC34A2-ROS1	L2155S	Protein dysfunction	lorlatinib
			cabozantinib
			repotrectinib
EZR-ROS1	S1986F/Y	Increased kinase activity	lorlatinib
			cabozantinib
			repotrectinib

#### 7.1.2 Activation of other signaling pathways

Crizotinib resistance may also be associated with the activation of other signaling pathways downstream of *ROS1*. The activation of these downstream signaling pathways can lead to the following: 1) stimulation of the signaling pathway that resists ROS1-TKIs and 2) the production of new mutations or expansions at the level of other oncogenes. For example, SHP2 activation of the MAPK/MEK/ERK pathway can lead to TKI resistance. In *in vitro* experiments, a combination of SHP2 inhibitors and ROS1-TKI increased the inhibition of tumor growth (Li et al., 2021). Additionally, mutations or proliferation of *ALK* (Li et al., 2021), *BRAF* (Ren et al., 2021), *KRAS*, and *MET* (Wang et al., 2021) can lead to resistance to ROS1-TKIs.

#### 7.1.3 Phenotype transformation

In a related case report, small-cell cancer transformation may occur after the resistance to ROS1-TKIs (Yang et al., 2021). According to these findings, this phenotype transformation may be associated with the inactivation of the retinoblastoma 1 (*RB1*) and *TP53* genes (Fares et al., 2020; Lin et al., 2020).

### 7.2 Resistance mechanisms of lorlatinib

The mechanisms underlying the resistance to TKIs other than crizotinib are not fully understood. Lin et al. analyzed 28 cases of post-lorlatinib progressive tumor tissue samples and found mutations in the kinase structural domain, especially *G2032K* and *L2086F* mutations (Lin et al., 2021). The results of *in vitro* experiments showed that the *ROS1* ^*G2032K*^ mutation conferred resistance to crizotinib, entrectinib, and lorlatinib. For the *ROS1* ^*L2086F*^ mutation, the related models showed that it was equally resistant to crizotinib, entrectinib, and lorlatinib, whereas cabozantinib may have a therapeutic effect (Mazieres et al., 2015). Other resistance mechanisms to lorlatinib include *MET* expansion (4%), *KRAS* ^*G12C*^ mutation (4%), *KRAS* expansion (4), and *NRAS* extension (4%) (Papadopoulos et al., 2020).

## 8 Conclusion

Targeted therapy is the foundation for the treatment of people with unresectable NSCLC with *ROS1* rearrangements. Crizotinib and entrectinib are currently recommended as the standard first-line therapeutic drugs. In future drug development, antitumor activity and brain permeation are important indicators for measuring the effectiveness of ROS1-TKIs. The new TKIs, entrectinib and lorlatinib, have a higher rate of blood–brain barrier permeation and are expected to provide increased control of brain metastases. For the primary mutants of *ROS1* ^*G2302R*^, repotrectinib and taletrectinib also showed high clinical efficacy.

In cases of disease progression after crizotinib treatment, the choice of secondary treatment should depend on the type of progression and specific resistance mechanisms. In the case of oligometastasis, topical treatment, represented by radiation or surgery, should be quickly administered, especially in patients with brain metastases. When multiorgan progression occurs, a whole-body treatment scheme based on chemotherapy containing platinum is still the current standard treatment. Targeted drugs may be more suitable treatment options for patients who have undergone genetic testing to clearly identify resistance mechanisms.
